# Prevalence Study of Dermatologic Manifestations among Diabetic Patients

**DOI:** 10.1155/2019/5293193

**Published:** 2019-07-01

**Authors:** Zahra Azizian, Elham Behrangi, Roshanak Hasheminasabzavareh, Hassan Kazemlo, Roja Esmaeeli, Parvaneh Hassani

**Affiliations:** ^1^Iran University of Medical Sciences, Tehran, Iran; ^2^College of Engineering, University of Alzahra, Tehran, Iran; ^3^Islamic Azad University, Tehran Medical Sciences Branch, Tehran, Iran

## Abstract

**Background:**

Diabetes mellitus (DM) is an interdisciplinary disorder that needs many different specialties' attention; however, the importance of dermatologists' knowledge has not been highlighted regarding this issue. As a result, we aim to assess the prevalence and variety of DM skin and nail manifestations in an effort to further acquaint dermatologists and other clinicians with diabetic dermatologic manifestations.

**Methods:**

This was a cross-sectional study; subjects who had a diagnosis of DM, attending to the outpatient endocrinology clinics of Rassoul Akram Hospital, Tehran, Iran, were evaluated by one dermatologist for diabetic dermopathy. The results were recorded in prepared data sheets, and general descriptions of DM duration, DM type, DM control, and drug history as well as the demographic data were gathered.

**Results:**

Among a total of 255 subjects, the prevalence of one or more identifiable skin conditions was 88.4%. 15.7% of the subjects had nail manifestations. Among cutaneous manifestations, acanthosis nigricans, acral erythema, and onychoschizia showed a significant relationship with the age and disease duration (p<0.05); and knuckle pebbles, eczema, facial erythema, and koilonychias had a meaningful relationship with FBS level and glycated hemoglobin, HbA1c (p<0.05).

**Conclusion:**

This study provides an overview that facilitates earlier detection and treatment of DM. Also, this data can help physicians and endocrinologists to visualize DM control level.

## 1. Introduction

Diabetes mellitus (DM) is the most common endocrine disorder with a significant burden on the patients, health care system, and the society [1, 2]. About 11 million people in the USA are diagnosed with DM, of which 90% are insulin-independent DM [3]. Some cutaneous manifestations related to DM such as acanthosis nigricans and pigmented purpuric dermatosis are the signs of macrovascular complications [4]. At least 30% of patients with DM are affected by different types of cutaneous disorders during the chronic course of their disease [5]. In the classification of cutaneous manifestations in DM, they are divided into four categories: (1) cutaneous diseases with weak to strong association with DM; (2) cutaneous infections; (3) cutaneous manifestations of DM complications; and (4) cutaneous reactions to DM treatments [6, 7]. Long-term DM duration causes permanent and irreversible functional changes and damage to body cells, and therefore, it leads to problems arising from biochemical, structural, and functional anomalies [8, 9]. Cutaneous complications of DM provide a clue to the current and past metabolic status of the patient [5]. Cutaneous infections occur in 20- 50% of patients and are often along with moderate blood glucose control. Microvascular circulatory disorders, peripheral vascular diseases, peripheral neuropathy, and immune responses reduction are all contributing factors to an increased susceptibility of infection [10]. Common cutaneous infections, staphylococcal infections, are more perilous and severe in patients with uncontrolled DM. Other types of infection include styes that cause tuberculosis of eyelid and also bacterial infection of the nails [11]. A fungus called* Candida albicans* is responsible for numerous fungal infections affecting diabetic patients; these infections are common in vaginal area and lips corners (angular cheilitis) [11]. Candidiasis infection (moniliasis) can be considered as an early symptom of undiagnosed DM and localized candidiasis infection in the genital area of women has a strong relationship with DM [12].

Increasing the knowledge about cutaneous manifestations of DM can be associated with overall prognosis improvement of disease through the early diagnosis and treatment [13]. According to various studies, 30-82% of DM patients experience different types of cutaneous disorder during the chronic course of their disease [6, 14]. Controlling the metabolism of the body may prevent some of these manifestations and also support the treatment [15]. On the other hand, many glycemic control medications also have skin side effects [16]. People who have cutaneous manifestation related to DM, even without a history of DM, should be investigated for the possibility of the disease [17].

Diabetes mellitus (DM) is a highly prevalent interdisciplinary disorder that needs many different specialties' attention; however, the importance of dermatologists' knowledge has not been highlighted regarding this issue. As a result, we aim to assess the prevalence and variety of DM skin and nail manifestations in an effort to further acquaint dermatologists and other clinicians with diabetic dermatologic manifestations.

## 2. Materials and Methods

In this descriptive cross-sectional study, diabetic patients attending to the outpatient endocrinology clinics of Rassoul Akram Hospital were evaluated by the same dermatologist for diabetic dermopathy. All patients were then asked about any dermatological condition they might have and subsequently all were examined, regardless of their response. They underwent a full dermatological exam and screening under the supervision of an academic dermatologist during the years 2014 and 2015. The dermatological exam results were recorded in prepared data sheets, and general descriptions of DM duration, DM type, DM control, and drug history were gathered. The studied population consisted of 255 patients; this number was estimated using the formula for calculating sample size:

(1) $$\begin{array}{c} N=Z\left(\;1-\frac{\alpha }{2}\;\right)2pq*d-2. \end{array}$$

The data were then entered into SPSS software (SPSS for Windows, Rel. 10.0.0. 1999; Chicago: SPSS Inc.) for analysis. The normal distribution of quantitative variables was performed through one-sample Kolmogorov-Smirnov test and Levene's test. Among the quantitative variables of age, disease duration, fasting blood sugar (FBS) level, and hemoglobin A1c level (HbA1c), only the quantitative variable of age had parametric normal distribution (one-sample Kolmogorov-Smirnov test significance: 200.0). Therefore, for investigating the relationship of quantitative variables of age, disease duration, FBS level, and HbA1c level with cutaneous manifestations in patients, independent sample T-test was used for the variable of age, and Mann-Whitney U test was used for other variables. The P value less than 0.05 was considered significant.

## 3. Results

A total of 225 diabetic subjects were included. Among the 225 patients, 30.2% (n = 68) were male and the average age of the total subjects was 55.85 (±13.04). The descriptive summary of study sample is shown in Table 1 (Table 1). Correlation of older age with history of diabetic nephropathy (P=0.004), retinopathy (P= 0.001), and neuropathy (P=0.00) was significant. But the correlation of older age and history of autoimmune disease was not significant (P=0.1). Correlation of longer duration of the disease with history of diabetic nephropathy (P value 0.003), retinopathy (P=0.0), neuropathy (P=0.01), autoimmune disease (P=0.007), hypothyroidism (P=0.002), and rheumatoid arthritis (P=0.03) was significant. History of diabetic retinopathy was significantly correlated with higher mean blood sugar (P=0.03) and higher HbA1c (P=0.01).

**Figure 1:**
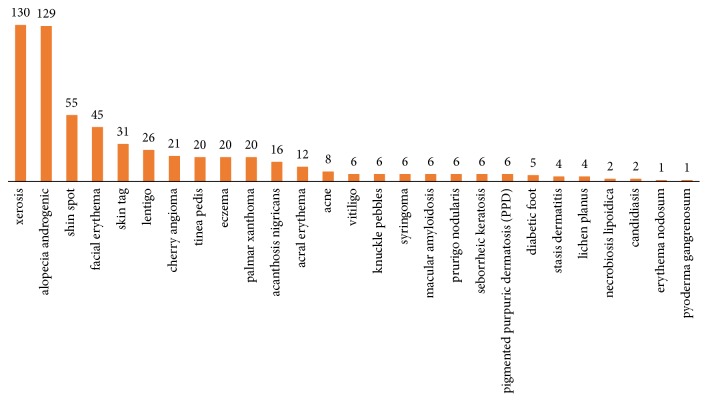
Cutaneous and nail manifestations in studied diabetic patients in the research in terms of the prevalence rate.

**Figure 2:**
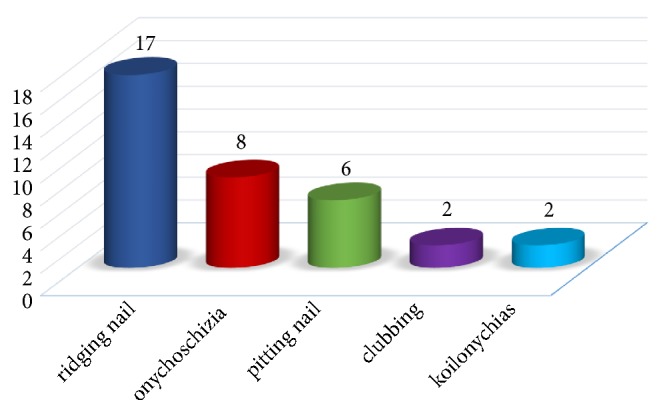
Cutaneous manifestations related to nail in studied diabetic patients in terms of the prevalence rate.

The overall prevalence of one or more (1 to 7) identifiable skin conditions was 88.4% (Figure 1). 15.7% of the patients had nail manifestations. Ridging nail was the most common one which was detected in 17 cases (7.6%) followed by onychoschizia in 8 patients (3.6%), pitting nail in 6 patients (2.7%), clubbing in 2 patients (0.9%), and koilonychias in 2 patients (0.9%) (Figure 2).

Amongst cutaneous manifestations, acanthosis nigricans, acral erythema, and onychoschizia presented a significant relationship with age and disease duration. Knuckle pebbles, eczema, facial erythema, and koilonychia showed a significant association with FBS level and glycated hemoglobin, HbA1c (p<0.05) (Table 2).

## 4. Discussion

The purpose of this study was assessing the prevalence and variety of DM skin and nail manifestations to further acquaint dermatologists and other clinicians with diabetic dermatologic manifestations. The results of this study confirmed our hypothesis that identifiable skin conditions have a very high prevalence amongst diabetic patients.

In our sample of diabetic patients, DM type 2 was more prevalent like most of the random diabetic patients' samples [18]. Our study subjects were about 20 percent overweight with mean body mass index (BMI) of 27 kg/m2. Obesity is defined as BMI of ≥30 kg/m2 and is prevalent among diabetics, since it can predispose to metabolic syndrome, one of the risk factors of DM type 2. Literature has shown the effect of obesity on DM and even on diabetic cutaneous manifestations but this effect does not become significant under the BMI of 30 kg/m2 such as our study sample [19]. Among the systemic complications of DM, neuropathy, retinopathy, and nephropathy showed the highest prevalence, respectively, in our subjects and 25% had at least one of these three disorders. Cutaneous manifestations were generally observed in 88.4%; there is thus a relative consistency between our study and previous studies in this domain [12, 20]. In the study of Chatterjee et al. 67% of subjects showed more than one cutaneous manifestation [21]. In our study 76% of patients had more than one cutaneous manifestation. A review of different researches indicates a wide variety of reported prevalence for different types of cutaneous manifestations in diabetic patients. Xerosis and Androgenic Alopecia were two main common cutaneous disorders detected among our patients. Skin tag, facial erythema, shin spot, and lentigo were, respectively, the most prevalent cases afterwards. Ridging nail (7.6%) was the most common nail manifestation detected. In a research conducted by Goyal et al. on 100 diabetic patients, Xerosis was reported as the most common cutaneous manifestation with a prevalence of 44% and was followed by skin dermopathy and diabetic tags [20]. Cutaneous infections and diabetic dermopathy were the most common cutaneous manifestations in the study of Furqan et al. in 2014 on 100 diabetic patients with DM types 1 and 2 [20]. Infections, Xerosis, below knee hair loss, and diabetic dermopathy were the most common cutaneous manifestations in the study of Chatterjee et al. and in the study of Vahora et al. on 300 diabetic patients; infections and acanthosis nigricans were reported as the most common cutaneous manifestations [2, 21]. Ragunatha et al. showed signs of insulin resistance, and fungal and bacterial infections are the most common manifestations [20].

The older age and longer DM duration had a meaningful relationship with diabetic retinopathy, nephropathy, and neuropathy. The average age and disease duration in patients with these disorders were significantly higher than in nonaffected patients. Among cutaneous manifestations, acanthosis nigricans, acral erythema, and onychoschizia showed a significant relationship with age and disease duration; and knuckle pebbles, eczema, facial erythema, and koilonychia had a meaningful relationship with fasting blood sugar level and hemoglobin HbA1c. Chatterjee et al. conducted a study to investigate the prevalence and pattern of skin disorders on 680 diabetic patients; they did not find a statistically significant relationship between the occurrence of cutaneous diseases and metabolic glucose control [21]. In our study, only cutaneous manifestations of acral erythema, acanthosis nigricans, and onychoschizia showed a significant relationship with the age and disease duration; this fact may also indicate that most DM cutaneous manifestations are not associated with the age and disease duration. In the descriptive study by Ragunatha et al. in 2011 with the purpose of investigating the effect of DM control on the occurrence of cutaneous manifestations in 500 diabetic patients, there was no statistically significant difference in patients in terms of age, gender, DM duration, and fasting plasma glucose [18]. However, in the study of Chatterjee et al., a statistically significant relationship was reported between cutaneous manifestations and DM duration [21]. Also, according to the results of a study conducted by Shahzad et al. on 320 diabetic patients, in patients with less than 5-year DM duration, the prevalence of cutaneous manifestations was 80%, whereas in patients with more than 5-year duration, the prevalence of cutaneous manifestations was 98%, and this difference was statistically significant (p<0.001) [17].

## 5. Conclusion

DM is a common endocrine disorder that frequently accompanies skin manifestations—up to 80% of patients with DM are affected. Recognition of the clinical features of DM is critical, as delayed detection and untreated disease may result in a significantly reduced quality of life. DM is also associated with cardiometabolic and cerebrovascular comorbidities, including coronary heart disease, hypertension, dyslipidemia, and cerebrovascular accidents, further highlighting the importance of identifying affected patients. Dermatologists are poised for the early detection of skin manifestations of DM, as diabetic ulcers predate its development in many of patients. Until recent decades diabetic foot ulcer was the only diabetic skin manifestation that attracted authors' notification. This study shows the prevalence and variety of DM skin and nail manifestations in an effort to further acquaint dermatologists and other clinicians with diabetic dermatologic manifestations. This study provides an overview that may facilitate earlier detection and treatment of the disorder. Also, this matter can help physicians and endocrinologists to have a visualized idea of DM control and situation.

## Figures and Tables

**Table 1:** Descriptive summary of study sample.

Study variable	Descriptive Statistics
Age (mean ± SD)	55.85 ± 13.04

Gender (n (%))	
Male	68(30.2%)
Female	157(69.8%)

BMI (mean ± SD)	27±3

DM (n (%))	
Type 1	9(4%)
Type 2	216(96%)

DM duration (mean ± SD)	8.06 ± 7.16

Fasting Blood Sugar	155.64 ± 53.16

HbA1c (mean ± SD)	7.77 ± 1.66

Type of treatment (n (%))	
Insulin	59 (26.2%)
Drug treatment	156 (69.3%)
Without treatment	10 (4.4%)

Associated diseases (n (%))	
Autoimmune diseases	28 (12.4%)
Hypothyroidism	20 (8.9%)
Rheumatoid arthritis	2 (0.9%)

Main associated clinical symptoms (n (%))	
Retinopathy	31 (13.8%)
Nephropathy	16 (7.1%)
Neuropathy	58 (25.8%)

**Table 2:** Association of DM related cutaneous and nail manifestations with DM duration.

Manifestation	Mean duration of the disease in patients (year)		P-value
	Positive	Negative	
Acral erythema	16.13	7.61	0.035
Skin tag	7.10	8.22	0.038
Acanthosis nigricans	3.46	8.42	0.001
Onychoschizia	3.20	8.24	0.021

## Data Availability

The data used to support the findings of this study are included within the article.
